# Nanoparticle‐Mediated Intracellular Protection of Natural Killer Cells Avoids Cryoinjury and Retains Potent Antitumor Functions

**DOI:** 10.1002/advs.201902938

**Published:** 2020-03-13

**Authors:** Xue Yao, Joshua J. Jovevski, Michaela F. Todd, Rui Xu, Yining Li, Jiao Wang, Sandro Matosevic

**Affiliations:** ^1^ Department of Industrial and Physical Pharmacy Purdue University West Lafayette IN 47907 USA; ^2^ Center for Cancer Research Purdue University West Lafayette IN 47907 USA

**Keywords:** DMSO‐free cryopreservation, immunotherapy, nanoparticle‐based cryopreservation, natural killer cells

## Abstract

The ability of natural killer (NK) cells to mediate potent antitumor immunity in clinical adoptive transfer settings relies, in large part, on their ability to retain cytotoxic function following cryopreservation. To avoid potential systemic toxicities associated with infusions of NK cells into patients in the presence of dimethylsulfoxide (DMSO), interest in alternative cryoprotective agents (CPAs) with improved safety profiles has grown. Despite the development of various sugars, amino acids, polyols, and polyampholytes as cryoprotectants, their ability to promote protection from intracellular cryodamage is limited because they mostly act outside of the cell. Though ways to shuttle cryoprotectants intracellularly exist, NK cells' high aversity to manipulation and freezing has meant they are highly understudied as targets for the development of new cryopreservation approaches. Here, the first example of a safe and efficient platform for the intracellular delivery of non‐DMSO CPAs to NK cells is presented. Biocompatible chitosan‐based nanoparticles are engineered to mediate the efficient DMSO‐free cryopreservation of NK cells. NK cells cryopreserved in this way retain potent cytotoxic, degranulation, and cytokine production functions against tumor targets. This not only represents the first example of delivering nanoparticles to NK cells, but illustrates the clinical potential in manufacturing safer allogeneic adoptive immunotherapies “off the shelf.”

## Graphical abstract

The clinical use of natural killer (NK) cells in immunotherapy requires cryopreservation prior to administration to patients. The process of cryopreservation, however, damages NK cells and, when DMSO is used, risks severe toxicities. Here, the development of a safe new nanoparticle‐based approach is described for the intracellular cryoprotection of NK cells, which yields functional effectors post cryopreservation devoid of DMSO.

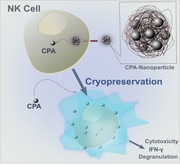

## 1 Introduction

Though natural killer (NK) cells have shown remarkable antitumor responses in patients with a variety of malignancies, significant challenges to their use in adoptive transfer immunotherapies remain.^[^ ^1^ ^]^ Among them, cryopreservation is considered a notable burden to the safe use of these cells clinically.^[^ ^2^ ^]^ Dimethylsulfoxide (DMSO), the most commonly used cryoprotectant, is widely ubiquitous largely due to its efficiency and lack of equivalent alternative.^[^ ^3^ ^]^ However, it has been associated with severe toxicities when infused into patients.^[^ ^4^, ^5^, ^6^, ^7^, ^8^, ^9^, ^10^, ^11^, ^12^, ^13^ ^]^ Adverse effects of DMSO have also been studied extensively in vitro, with studies showing DMSO to be toxic to various cell types including blood cells,^[^ ^14^, ^15^ ^]^ mesenchymal stromal cells,^[^ ^16^ ^]^ skin fibroblasts,^[^ ^17^, ^18^ ^]^ and human corneal endothelial cells.^[^ ^19^ ^]^ DMSO also has the ability to induce unwanted epigenetic changes.^[^ ^20^, ^21^, ^22^ ^]^

Studies have shown that while NK cells are generally able to tolerate cryopreservation, specific treatment conditions are necessary to maximize recovery and function post‐thaw. Resting, for instance, the cells after thawing has been reported to restore cytotoxic functions of NK cells otherwise impaired following cryopreservation,^[^ ^23^, ^24^, ^25^, ^26^, ^27^ ^]^ including degranulation and killing capacity.^[^ ^28^ ^]^ In addition, cytokines are often needed for NK cells to regain function after being frozen, particularly IL‐2 and IL‐15.^[^ ^29^ ^]^ Incubating thawed NK cells with IL‐2 for 16 h was, for instance, shown to reverse the cryopreservation‐induced loss in expression of activating receptors TRAIL (TNF‐related apoptosis‐inducing ligand) and NKG2D on NK cells and a corresponding reduction in cytolytic ability.^[^ ^30^ ^]^

Effects on NK cell responses are likely protocol specific. When cryopreserved with DMSO, NK cells from hematopoietic stem cell transplantation (HSCT) recipients were reported to show elevated expression of a functionally impaired CD56^dim^CD16^−^ subset,^[^ ^31^ ^]^ a phenotype not present on NK cells from fresh peripheral blood. Whether this change was induced due to DMSO in freezing media, the cryopreservation process or both is not clear. Other studies have also reported impairment in cytotoxicity of NK cells compared to that of fresh, non‐cryopreserved NK cells after cryopreservation either in vitro^[^ ^32^ ^]^ or following adoptive transfer of NK cells into immunodeficient tumor‐bearing mice.^[^ ^27^ ^]^ Conversely, other studies reported no functional impairment of NK cells following cryopreservation.^[^ ^33^ ^]^ Torelli et al.^[^ ^34^ ^]^ showed that thawed NK cells that had been expanded ex vivo with IL‐2 and IL‐15 following cryopreservation with human serum albumin (HSA) and DMSO were able to degranulate and kill K562 target cells as effectively as fresh NK cells.

What most of these studies have in common is the presence of DMSO. In response to its reported toxicities,^[^ ^35^ ^]^ alternative cryoprotective agents (CPAs) have been evaluated. They include sugars, amino acids, polyols, polyampholytes, and various polymers in combination or individually.^[^ ^36^ ^]^ Among these, trehalose has been among the most widely used, owing to its favorable cryoprotective properties.^[^ ^37^, ^38^ ^]^ However, unlike DMSO, trehalose is unable to cross the cell membrane, limiting its potential utility,^[^ ^39^, ^40^, ^41^ ^]^ while mammalian cells are unable to synthesize it.^[^ ^42^ ^]^ If cells are cooled too rapidly, they are unable to dehydrate before intracellular water freezes, forming growing ice crystals that have the potential to cause severe cryoinjury. Slow cooling rates, on the other hand, lead to osmotic imbalance as water leaves the cell, which is also damaging to cells.^[^ ^43^ ^]^ This not only implies that cooling rates have to be carefully selected for each cell type, but that intracellular protection of cells from the freezing process is critical in maintaining their integrity and viability. To meet the need for trehalose to be present on both sides of the cell membrane,^[^ ^44^, ^45^ ^]^ strategies to shuttle it across the membrane have included nanoparticles,^[^ ^41^ ^]^ electroporation,^[^ ^46^ ^]^ macrocycles,^[^ ^47^ ^]^ freezing,^[^ ^48^ ^]^ and liquid‐phase endocytosis.^[^ ^49^ ^]^ Though attractive, none of these approaches have been attempted with NK cells, likely because of their high aversity to any kind of exogenous manipulation, requiring meticulous process development.

We recently reported the cryopreservation of NK cells with combinations of non‐DMSO CPAs.^[^ ^50^, ^51^ ^]^ While our results show that NK cells cryopreserved under these conditions retain cytotoxic functions, none of these CPAs are able to penetrate the cell membrane, limiting potential intracellular damage that NK cells experience during the freezing and thawing process, and impairing durable functional responses.

Here, we describe the first example, to our knowledge, of the development of a simple and biocompatible nanoparticle‐based platform for the intracellular delivery of non‐DMSO CPAs to NK cells for use in immunotherapy. By promoting intracellular protection of NK cells, this approach yields functional NK cells that are able to mediate efficient antitumor activity against tumor targets. This is the first example of the feasibility of internalizing CPAs by NK cells, ultimately limiting freezing‐induced damage which can occur as a result of the intracellular ice crystal formation, and enabling cryopreservation to occur in the presence of simple, biocompatible CPAs. More generally, this is the first example of the demonstration that nanoparticle‐mediated delivery to NK cells is a potentially viable strategy to achieve modulation of their cellular responses.

## 2 Results

### 2.1 Size and Surface Charge of CS:TPP Nanoparticles

**Figure 1:**
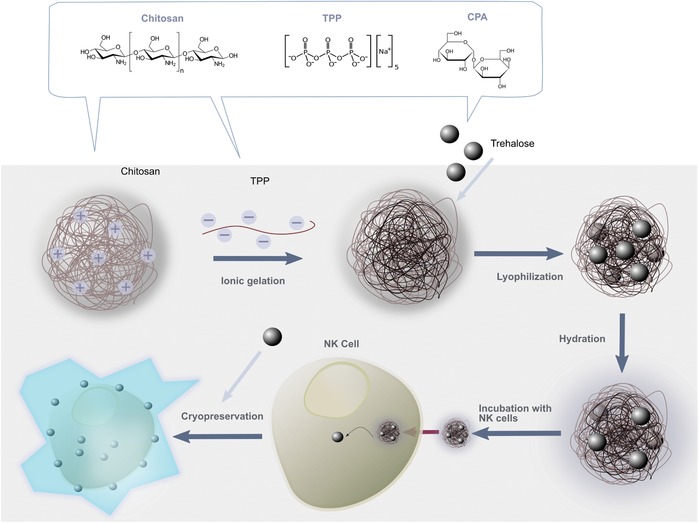
Assembly of nanoparticles and delivery to natural killer cells. Chitosan–TPP nanoparticles are assembled via ionic gelation. CPAs (for example, trehalose) are loaded inside the nanocarriers and the nanoparticles are lyophilized, prior to being rehydrated in cell culture media. Delivery to NK cells occurs by incubating the CPA‐loaded nanoparticles with NK cells at 37 °C. This promotes internalization of the nanoparticles and the intracellular delivery of CPA cargo to NK cells. Following nanoparticle internalization, NK cells are able to be cryopreserved at subzero temperatures in liquid N_2_ in the absence of DMSO. Thawed NK cells are then used for adoptive transfer immunotherapy.

**Figure 2:**
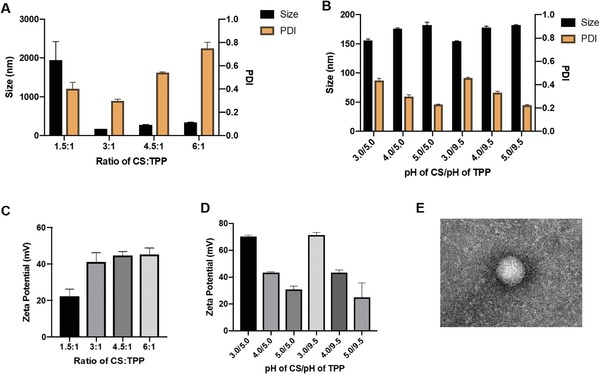
Assembly and characterization of chitosan–TPP nanoparticles. A) Effect of different ratios of chitosan and TPP on the size and PDI of the nanoparticles. B) Effect of pH of chitosan and TPP on size and PDI of nanoparticles. The pH of chitosan was varied between 3 and 5, while the pH of TPP was either 5 or 9.5. C) Zeta potential of nanoparticles at different chitosan:TPP ratios. D) Zeta potential of nanoparticles as a function of pH of chitosan and TPP at a chitosan:TPP ratio of 3:1. The pH of chitosan was varied between 3 and 5, while the pH of TPP was either 5 or 9.5. E) TEM image of nanoparticle. Scale bar: 100 nm.

To achieve internalization of CPAs by NK cells (**Figure** **1**), nanoparticles of chitosan (CS)–tripolyphosphate (TPP) were synthesized. Positively charged chitosan is able to form nanoparticles with negatively charged TPP by ionic gelation.^[^ ^52^ ^]^ Nanoparticles were prepared at various ratios of chitosan and TPP and characterized in terms of size, diameter, polydispersity index (PDI), and surface charge. As shown in **Figure** **2**A–D, by increasing the weight ratio of CS:TPP from 3:1 to 6:1, the size and the PDI of nanoparticles also increased. Specifically, the mean diameter increased from 178.56 ± 1.53 nm (at 3:1) to 344.28 ± 16.13 nm (at 6:1) while the PDI changed from 0.297 ± 0.029 to 0.748 ± 0.093. Given these observations on the effect of CS and TPP on average nanoparticle size and PDI, nanoparticles with most favorable size (<200 nm) and monodispersity could be obtained at a CS:TPP ratio of 3:1. It has to be noted that at a CS:TPP ratio of 1.5:1, the size of the nanoparticles dramatically increased to >2000 nm, likely due to particle sedimentation. Similarly to size and PDI, the surface potential of the nanoparticles increased in response to changing ratios of CS and TPP, ranging from +22.29 ± 4.003 mV (at 1.5:1) to +45.27 ± 3.611 mV (at 6:1; Figure 2C).

We also measured the effect of pH both on the size and surface potential of nanoparticles. As Figure 2B shows, at a pH of TPP of either 5.0 or 9.5, an increase of the pH chitosan pH from 3.0 to 5.0 led to a corresponding increase in the mean size diameter but a decrease in PDI. The surface charge showed a similar trend as the PDI. The pH of TPP, on the other hand, did not show any appreciable effect on nanoparticle physical properties. In summary, nanoparticles with the smallest size, optimal PDI, and high surface potential were obtained at a 3:1 ratio of CS:TPP at a pH of either 5.0 or 9.5. We selected these conditions for further studies.

### 2.2 Morphological Characteristics of CS–TPP Nanoparticles

Transmission electron microscope (TEM) imaging was carried out to visually characterize the morphology of the assembled nanoparticles. As shown in Figure 2E, nanoparticles were spherical in shape with a diameter ranging between 100 and 200 nm, which was consistent with our results obtained by dynamic light scattering at the nanoparticle assembly conditions selected.

### 2.3 Encapsulation of Trehalose Inside CS–TPP Nanoparticles and Their Characterization

**Figure 3:**
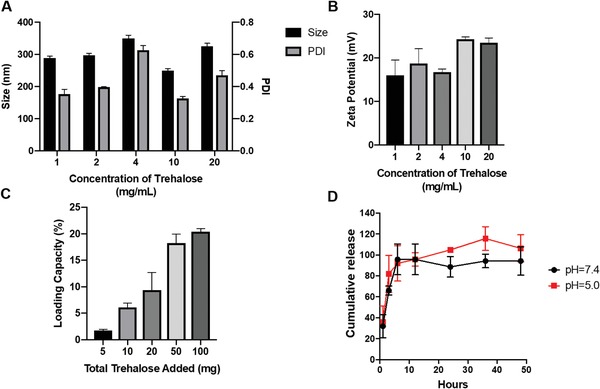
Encapsulation of trehalose inside chitosan–TPP nanoparticles. All nanoparticles were prepared at a chitosan:TPP ratio of 3:1. A) Effect of amount of loaded trehalose on the size and PDI of chitosan–TPP. The concentration of trehalose ranged from 1 to 20 mg mL^−1^. B) Zeta potential of nanoparticles at different amounts of trehalose, ranging from 1 to 20 mg mL^−1^. C) Loading capacity of chitosan–TPP nanoparticles encapsulating trehalose. The loading capacity was determined at various amounts of loaded trehalose ranging from 5 to 100 mg. D) Release of trehalose from chitosan–TPP nanoparticles. The amount of trehalose released was measured by dialysis at pH values of 5.0 and 7.4 over a period of 48 h.

Encapsulating trehalose inside CS:TPP nanoparticles enables it to be shuttled across the NK cell membrane, allowing for its intracellular presence where it can induce cryoprotection.^[^ ^53^ ^]^ To identify the maximum amount of trehalose that can be encapsulated inside the CS:TPP nanoparticles, various concentrations of trehalose ranging from 1 to 20 mg mL^−1^ were directly dissolved in the TPP solution. Using a CS:TPP ratio of 3:1 and corresponding pH of 5.0 for chitosan and 9.5 for TPP, different concentrations of trehalose/TPP solution were added into the chitosan solution drop wise to assemble the encapsulated nanoparticle (trehalose‐loaded nanoparticle, nTre). As shown in **Figure** **3**A, 10 mg mL^−1^ trehalose yielded nTre with a size of 250.18 ± 10.609 nm and a PDI of 0.325 ± 0.023. Surface zeta potential was also measured (Figure 3B), with 10 mg mL^−1^ trehalose exhibiting a higher zeta potential (+24.28 ± 0.956 mV) compared to other concentrations. Although the particle size increased after loading trehalose, the surface zeta potential did not show any significant change. Loading capacity (Figure 3C) was determined as the ratio of the weight percentage of trehalose in nanoparticles from the total weight of both trehalose and nanoparticles. Increasing the total amount of trehalose was associated with higher loading capacities, which demonstrated that trehalose can be encapsulated into nanoparticles more efficiently.

### 2.4 Release of Trehalose from CS:TPP Nanoparticles

To determine the stability of nTre, we performed a spontaneous release assay at pH 5 and 7.4 to simulate a physiological environment (Figure 3D). Trehalose could be released from CS:TPP nanoparticles at both pH values, achieving almost complete release after 6 h. The release of trehalose from CS:TPP nanoparticles was slightly faster at pH 5 than at pH 7 during the first 6 h, indicating that the acidic pH may facilitate its release into the aqueous phase. Considering the intracellular environment of the endosome and lysosome is highly acidic, we can assume that trehalose could be released from the nanoparticles successfully within 12 h. We further confirmed, by lysing NK cells after incubation with nTre, that they were able to release trehalose indicating the successful internalization of trehalose with no premature loss of trehalose from the cell interior, contrary to free trehalose controls (Figure S2, Supporting Information).

### 2.5 Effect of Nanoparticles on the Viability of NK Cells

**Figure 4:**
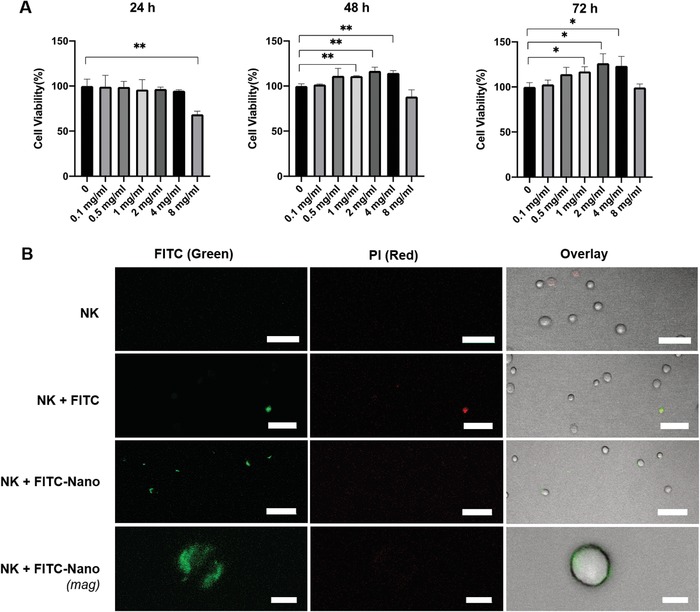
Uptake of trehalose‐loaded nanoparticles by NK cells. A) Viability of NK‐92 cells in the presence of chitosan–TPP nanoparticles. Cells were incubated with various concentrations of nanoparticles, ranging from 0.1 to 8 mg mL^−1^, at 37 °C in culture media, and the viability of the cells measured over a period of 72 h. B) Confocal imaging of uptake of chitosan:TPP nanoparticles by NK cells. Nanoparticles were loaded with FITC (green), and the cells were stained with propidium iodide (PI, red) to distinguish dead cells. NK cells alone showed no uptake of either nanoparticles of red fluorescence due to PI. Uptake of free FITC (NK + FITC) is seen only for cells which appear to also show red fluorescence, while NK cells incubated with nanoparticles (NK + FITC‐nano) show green fluorescence with no red fluorescence, indicating nanoparticle internalization. Scale bars: 50 µm (top three rows) and 10 µm (bottom row); *p < 0.05, **p < 0.01.

Having successfully generated nanoparticles that can encapsulate trehalose, we sought to determine the toxicity of nTre to NK cells. To do so, various concentrations of nTre were dissolved in phosphate‐buffered saline (PBS) and incubated with NK cells for 24, 48, and 72 h. None of the conditions showed any significant detrimental effect of nTre on the viability of NK cells (**Figure** **4**A). NK cells retained excellent proliferative ability after incubation in the presence of nTre for 72 h compared to that of fresh, nontreated cells, at a concentration of 4 mg mL^−1^. Only when the concentration was increased to 8 mg mL^−1^, did we observe a slight reduction in cell viability after 12 h of incubation. However, this was reversed as the cell viability could be recovered following 72 h of incubation. These data indicate that CS:TPP nanoparticles encapsulating trehalose are nontoxic and well tolerated by NK cells and do not impair their viability.

### 2.6 NK Cell Uptake of CS:TPP Nanoparticles

To determine the ability of nanoparticles to be taken up by NK cells, fluorescein isothiocyanate (FITC)‐labeled chitosan was prepared according to previous methods.^[^ ^54^, ^55^ ^]^ The FITC‐labeled chitosan was then used to synthesize nanoparticles (FITC‐nano) as before. As shown in Figure 4B, free FITC could not permeate the NK cell membrane on its own. Dead cells, stained with propidium iodide (PI), showed strong uptake of FITC, likely because of damaged or leaky membranes. However, strong green fluorescence was observed when the cells were incubated with FITC‐nano, suggesting that FITC nanoparticles could be successfully taken up by NK cells. Images indicate that the internalized FITC nanoparticle could be localized to the cytoplasm of the cells, but not the nucleus. For its cryoprotective activity, trehalose does not have to be confined to a specific subcellular location,^[^ ^53^ ^]^ confirming that the observed uptake results of FITC‐nano by NK cells are indicative of potential biological relevance. Nuclear staining of NK cells revealed that the nanoparticles were localized to the cytoplasm of the cell (Figure S3, Supporting Information).

### 2.7 The Effect of nTre in the Cryopreservation of NK Cells

**Figure 5:**
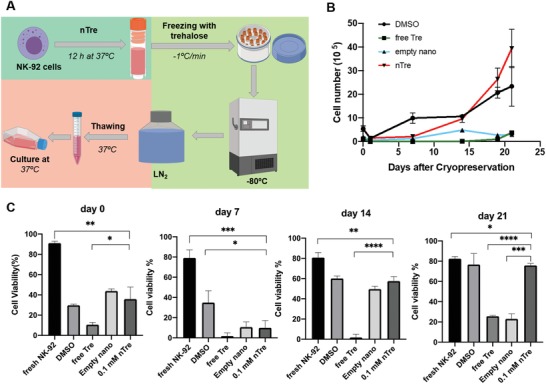
Nanoparticle‐mediated cryopreservation of NK cells. A) Diagram showing cryopreservation workflow followed in this work. B) Number of recovered NK cells in culture following cryopreservation. NK cells were pretreated with free trehalose alone, empty nanoparticles, trehalose‐loaded nanoparticles, or DMSO control. After cryopreservation and thawing in the absence of DMSO, NK cells were placed in culture media at 37 °C, and cell number was monitored over 21 days. C) Viability of NK cells in culture after cryopreservation. After thawing, cells from all treatment groups were placed in culture media at 37 °C and 5% CO_2_, and the viability measured by trypan blue staining, either immediately upon thawing (day 0), or after 7, 14, or 21 days; *p < 0.05, **p < 0.01, ***p < 0.001, ****p < 0.0001.

For the cryopreservation studies, we designed the freezing protocol shown in **Figure** **5**A. Briefly, NK cells were pretreated with empty nanoparticles or nTre for 12 h. The incubation time was selected based on the results obtained from the release and cellular uptake assays. After pretreatment, cells were collected and cryopreserved with trehalose freezing medium. Untreated NK cells were frozen in control freezing medium (50% fetal bovine serum (FBS) + 40% American Type Culture Collection (ATCC) medium + 10% DMSO) or free trehalose freezing medium. For all experimental groups, NK cells were cryopreserved in liquid nitrogen by slow freezing. After 3 days, cells in each group were thawed and cell number and viability were measured (Figure 5B,C). While NK cells cryopreserved with DMSO showed a cell recovery, including survival, comparable to nTre immediately and shortly after thawing (Figure S4A, Supporting Information), NK cells from the nTre group eventually exceeded the post‐thaw responses of DMSO and other groups. Free trehalose and empty nanoparticles did not show any cryoprotective effect to NK cells after thawing, as indicated by the poor viability throughout the entire post‐thaw period. Cell viability results were consistent with the NK proliferative data as shown in Figure 5C. Cell viability immediately after thawing ranged from 29.72% to 43.78% for the DMSO, empty nanoparticle, and nTre groups, while for the free trehalose group only 10.52% NK cells remained viable. Notably, 24 h after thawing, NK cell viability decreased rapidly for all groups (Figure S4B, Supporting Information), an observation consistent with our and other labs' previous studies.^[^ ^27^ ^]^ Interestingly, on day 14, NK cells from the nTre and DMSO groups showed comparable viabilities (DMSO: 60.13%; nTre: 57.51%). After 21 days, NK cells from both groups reached 75.91% and 76.69% viability, respectively, indicating that nTre‐cryopreserved NK cells are able to fully recover after cryopreservation. On the other hand, NK cells from the free trehalose group were mostly nonviable (25.61% viability) even on day 21, indicating that free trehalose alone was not sufficient to protect the cells during freezing and thawing and that intracellular protection is necessary. Similar trends were also observed with NK cell numbers over 21 days of culture post‐thaw (Figure 5B; Figure S4C, Supporting Information). Overall, these data suggest that pretreatment with nTre exerts a substantial and durable protective effect on NK cells during cryopreservation. nTre‐treated NK cells, moreover, show a superior proliferation compared to cells cryopreserved with DMSO.

**Figure 6:**
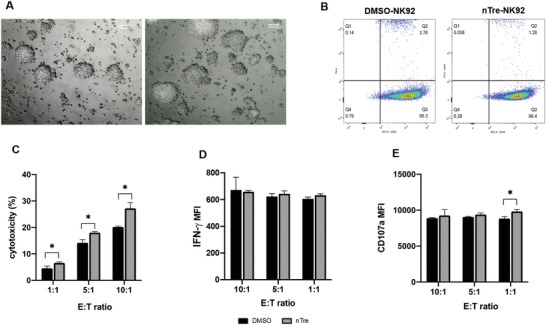
Cytotoxicity and functionality of NK cells after nanoparticle‐mediated cryopreservation. A) Morphology of NK cells in culture following cryopreservation. Cells that were cryopreserved after being loaded with trehalose‐encapsulating nanoparticles (left) showed similar morphology, upon expansion in cell culture media, to NK cells cryopreserved with DMSO (right). B) Quantitation of CD56^+^ NK cells by flow cytometry after cryopreservation for both nanoparticle‐treated NK cells and DMSO controls. C) Cytotoxicity of NK cells against K562 cells. Killing assays were carried at E:T ratios of 1:1, 5:1, and 10:1 for 4 h and killing of target cells was determined by 7‐AAD/carboxyfluorescein succinimidyl ester (CFSE) staining using flow cytometry. D) IFN‐γ production by NK cells when challenged to kill K562 cells. Same conditions as for the killing assay were used, and IFN‐γ expression was measured by flow cytometry. E) Degranulation of NK cells in response to K562 cells as measured by CD107a expression by flow cytometry; * < 0.05.

Morphologically, NK cells from both groups tended to grow in aggregates and form dense clumps during proliferation. There were no significant morphological differences observed between nTre and DMSO groups (**Figure** **6**A). We also examined the expression level of CD56 on cryopreserved NK cells. Over 98% of the population of nTre‐pretreated NK cells expressed CD56, similar to cells from the DMSO group (95.3% population; Figure 6B).

### 2.8 Functional Characterization of NK Cells After Freezing with nTre

NK cells possess the ability to mediate cytotoxicity against multiple malignancies, particularly K562 cells, a leukemic cell line, which does not express human leukocyte antigen (HLA) ligands so is unable to impair the cytotoxicity of NK cells. We measured the killing capacity of NK cells from the DMSO and nTre groups against K562 cells (Figure 6C). We observed increased cytotoxicity of NK cells from the nTre group against K562 cells at all three effector (NK cell):target (cancer cell) (E:T) ratios tested (1:1, 5:1, and 10:1). As expected, increasing the E:T ratio drives higher killing capacities. For the DMSO group, the average cytotoxicities were 4.59 ± 0.809% (1:1), 14.2 ± 1.21% (5:1), and 20.17 ± 0.38% (10:1), respectively. Compared to the DMSO group, NK cells in the nTre group mediated higher target cell killing—namely, 6.59 ± 0.38% (1:1), 18.03 ± 0.49% (5:1), and 27.2 ± 2.18% (10:1). Conventional cryopreservation with DMSO has been reported to impair the cytotoxicity of NK cells and our data here appear consistent with this, while Tre pretreatment could enhance the killing capacity of NK cells against target tumor cells.

It is known that activated NK cells are able to induce target cell apoptosis via contact‐dependent cytotoxicity following formation of an immunological synapse between NK cells and target cells. This is primarily mediated by degranulation and release of pro‐inflammatory cytokines such as interferon (IFN)‐γ.^[^ ^56^ ^]^ Therefore, to further elucidate the cytotoxic capacity of NK cells cryopreserved with nTre, we measured IFN‐γ and degranulation by CD107a against K562 cells. IFN‐γ expression from NK cells post‐thaw was comparable between the nTre and DMSO groups (for example, at the ratio of 1:1, mean fluorescence intensity for nTre group is 605.33 ± 14.012 while DMSO of 633 ± 10.583). Similar results were observed when degranulation was measured via CD107a expression (Figure 6C); independently of E:T ratio, no significant difference in degranulation between nTre and DMSO groups was detected. These data together indicate that NK cells cryopreserved with nTre are able to fully recover their proliferative potential and maintain their functionality, including cytotoxicity toward tumor cells, IFN‐γ production, and CD107a surface expression. In summary, our data indicate, for the first time, the potential therapeutic value of using nanoparticles to cryopreserve NK cells by inducing intracellular protection from cryodamage. We showed that CPAs could be delivered to NK cells intracellularly, without any impairment to their functional capacity.

## 3 Discussion

Administration of cell‐based immunotherapies to patients under centralized manufacturing models relies on a logistical workflow that makes cryopreservation a necessity. Though cryopreservation is not new, the rise in immunotherapies based on adoptively transferred immune cells, such as chimeric antigen receptor (CAR)‐modified NK cells,^[^ ^57^, ^58^ ^]^ has reignited the spotlight on the role of cryopreservation in the clinical preparation of these therapies. The loss of cell viability prior to infusion into patients is a recognized manufacturing hurdle for CAR‐T‐cell therapies.^[^ ^59^ ^]^ The reliance on 7.5% DMSO in the final formulation is another potential issue. Considering the recognized detrimental effects the process of thawing has on cells, the presence of apoptotic or necrotic cells in the infused product risks introducing potentially detrimental immunological responses.^[^ ^60^ ^]^

Unfortunately, not all cells respond to cryopreservation in the same way. Membrane permeability and composition, cell size, and surface‐to‐volume ratio are among factors which define individual cellular responses to freezing and thawing. For these reasons, cryoprotectants that have emerged as alternatives to DMSO—be it sugars, osmolytes, amino acids, or polyols—have not been able to replicate favorable responses seen with other mammalian cells when applied to the cryopreservation of immune cells. Though we recently showed that NK cells can be cryopreserved without DMSO,^[^ ^51^ ^]^ durable responses require cryoprotectants to be present on both sides of the cell membrane.^[^ ^61^ ^]^

In this study, we demonstrated that application of a simple carrier—chitosan‐based nanoparticles—is effective in inducing cryoprotection of NK cells by delivering the cryoprotectant trehalose intracellularly. Such intracellular protection, in turn, enables the retention of antitumor functions on NK cells following freezing and thawing in the presence of DMSO‐free CPAs. This platform is attractive because it is straightforward to manufacture and has been demonstrated as clinically safe.^[^ ^62^ ^]^ Our results show that pretreatment of NK cells with trehalose‐loaded nanoparticles successfully promoted the intracellular uptake of trehalose, which resulted in comparable viability and proliferation to using DMSO. Moreover, the recovered NK cells were able to retain mature functional activities, including cytotoxicity toward tumor cells, secretion of IFN‐γ, and degranulation. All these data suggest that delivering cryoprotectants into NK cells to reduce freezing‐induced cryoinjury is both feasible and practical. The retention of the full spectrum of functional NK cell antitumor activities may play a critical role in the future use of these cells as immunotherapies against multiple malignancies, including solid tumors.^[^ ^63^, ^64^ ^]^

Though a myriad of nanoparticle formulations of various complexities exist,^[^ ^65^ ^]^ our thrust was to advance NK cell cryopreservation without increasing the cost or complexity of this process. Chitosan–TPP nanoparticles fit these criteria; they are characterized by simple and mild preparation methods, favorable biodegradability, high biocompatibility, and low toxicity.^[^ ^66^ ^]^ Through a meticulous physicochemical optimization process, we identified conditions which enabled the assembly of nanocarriers that can efficiently deliver CPAs inside NK cells.^[^ ^40^, ^53^ ^]^ Using this platform, we were able to reduce the need for cryoprotectants to only trehalose, though using other CPAs such as those described in our previous studies is an obvious opportunity. This represents the first example, to our knowledge, of the use of nanoparticles to achieve any kind of intracellular delivery to NK cells and modulate their cellular responses.

Given its practical convenience, freeze–drying was used to preserve the trehalose‐encapsulated nanoparticles over long periods. However, freezing–drying overnight significantly increased the average sizes of nanoparticles due to aggregation and reduced their solubility (data not shown). To overcome this problem, lyoprotectants (varying concentrations of sucrose and mannitol) were added to the nanoparticles before freeze–drying. We found that mannitol (0.5–4% w/v concentrations) was superior to sucrose in generating workable nanocarrier formulations (Figure S1, Supporting Information).

The ability to use NK cells allogeneically speaks directly to the need for safer cryopreservation methods that do not rely on DMSO.^[^ ^67^ ^]^ In contrast to T cells, however, NK cells are more sensitive to freezing and thawing.^[^ ^2^ ^]^ This special sensitivity of NK cells to the cooling‐and‐warming process was reflected in our results (Figure 5C). After thawing, NK cells required cytokines (IL‐2) and a period of activation to regain full functionality. Nonetheless, cells from the nTre‐treated group ultimately showed significantly better proliferative properties, resulting in high proliferation rates in a relatively short time. These cells also displayed similar morphological properties, phenotype—via the expression of CD56—and comparable cytotoxicity to cells cryopreserved with DMSO. Assessment of the levels of expression of inhibitory and activating receptors following cryopreservation could be included in a future analysis.

In conclusion, this study provides the first example in the literature of delivering nanoparticles to NK cells, and shows that nanoparticle‐based approaches could promote the intracellular delivery of cryoprotectants into NK cells, ultimately leading to not only mature functional antitumor responses, but also the ability to safely manufacture allogeneic immunotherapies “off the shelf.” The improvements inherent within such an approach can bring us closer to a new clinical horizon in the development of NK cell‐based immunotherapies.

## 4 Conclusions

The use of NK cells in the treatment of complex cancers benefits from these cells' innate ability to recognize and kill pathogens. To advance the clinical attractiveness of NK cells as immunotherapeutic effectors, in this study we addressed two underexplored aspects of their use in immunotherapy: first, we achieved the first demonstration of the delivery on nanoparticles to NK cells, and, second, we demonstrated that biocompatible, easy‐to‐manufacture nanoparticle formulations are able to internalize a simple cryoprotectant into NK cells where it can significantly protect them against cryodamage while retaining a comprehensive spectrum of antitumor responses. Such DMSO‐free cryopreservation may signify a new path toward safe, efficient, and durable allogeneic cell‐based immunotherapies.

## 5 Experimental Section

##### Materials

Chitosan with low molecular weight (50–190 kDa based on viscosity, 75–85% deacetylated) and sodium tripolyphosphate were purchased from Sigma–Aldrich (St. Louis, MO, USA). d(+)‐trehalose dehydrate (99%) and FITC, isomer 1 (95%), were purchased from Alfa Aesar (Tewksbury, MA, USA). Trehalose assay kit was purchased from Megazyme (Wicklow, Ireland). Eagle's minimum essential medium (α‐MEM), Roswell Park Memorial Institute (RPMI)‐1640 medium, horse bovine serum, 2‐mercaptoethanol (2‐mer, 50 × 10^−3^ m), and penicillin and streptomycin were purchased from Gibco (ThermoFisher Scientific, USA). Myo‐inositol and folic acid were purchased from Sigma–Aldrich (St. Louis, MO, USA). FBS and DMSO were purchased from Corning (New York, NY, USA). Recombinant human IL‐2 (rhIL‐2) was gifted from Akron Biotech (Boca Raton, FL, USA). The CCK‐8 assay kit was purchased from Glpbio (Montclair, CA, USA). The CFSE/7‐AAD kit was purchased from Cayman Chemical (Ann Arbor, MI, USA). All the antibodies including PerCP‐Cy5.5‐conjugated IFN‐γ antibody (clone B27), phycoerythrin (PE)‐conjugated‐CD107a antibody (clone H4A3), GolgiStop solution, Cytofix/Cytoperm solution, and Perm/Wash buffer were purchased from BD Biosciences (San Jose, CA, USA). All other chemicals were purchased from Sigma, unless specifically noted otherwise.

##### Cell Lines

NK‐92 cells, obtained from the ATCC, were cultured in α‐MEM supplemented with 12.5% (v/v) horse bovine serum, 12.5% (v/v) fetal bovine serum, inositol (0.02 × 10^−3^ m), 2‐mercaptoethanol (0.1 × 10^−3^ m), folic acid (0.02 × 10^−3^ m), 100 U mL^−1^ penicillin, 100 µg mL^−1^ streptomycin, and 400 U mL^−1^ rhIL‐2. The human erythroleukemic cell line (K562) was obtained from ATCC and maintained in RPMI medium supplemented with 10% FBS, 100 U mL^−1^ penicillin, and 100 µg mL^−1^ streptomycin. All cell lines were incubated at 37 °C in a humidified 5% CO_2_ environment.

##### Synthesis of Chitosan/TPP Nanoparticle and FITC‐Labeled Nanoparticles

0.2% w/v chitosan (50–190 kDa based on viscosity) was dissolved in 2% (v/v) CH_3_COOH by stirring overnight at room temperature (RT) at a pH of 5.0, and filtered through a 0.22 µm filter. 0.1% w/v of sodium TPP was prepared by direct dissolution into ddH_2_O, with the pH adjusted to 9.5, followed by filtration (0.22 µm). To encapsulate trehalose inside chitosan/TPP nanoparticles, the trehalose/TPP mix was prepared by adding 1250 mg of trehalose into 50 mL of the TPP solution. Nanoparticles were prepared using the ionotropic gelation method between the molecular chains of chitosan and the polyanionic TPP ions under high agitation. About 4 mL of 0.1% TPP was added drop wise into 6 mL 0.2% chitosan under continuous magnetic stirring at 700 rpm for 30 min at room temperature. The pellet was subsequently collected by centrifugation at 20 000 rpm for 30 min at 4 °C and then resuspended into 5 mL of 4% mannitol solution (w/v, ddH_2_O). Nanoparticles were finally obtained after a freeze–drying step for 24 h.

FITC‐labeled nanoparticles (FITC‐NPs) for cellular uptake study were synthesized using FITC‐labeled chitosan according to a previously reported procedure.^[^ ^54^, ^55^ ^]^ FITC‐labeled chitosan was prepared by mixing 20 mL of FITC solution (1 mg mL^−1^ in dehydrated methanol) and 20 mL of chitosan solution (1%, w/v, in 0.1 m of CH_3_COOH). Following a reaction at room temperature in the dark for 3 h, FITC‐labeled chitosan was precipitated by adding NaOH. The precipitate was pelleted at 40 000 × *g* for 10 min and washed with methanol:water (70:30, v/v). The washing and centrifuging were repeated until no fluorescence was detected in the supernatant. The labeled chitosan was resuspended in 20 mL of 2% (v/v) CH_3_COOH and dialyzed (1000 Da molecular weight cutoff, MWCO) in the dark against 2 L of ddH_2_O for 24 h with the water being replaced with fresh water every 6 h. Finally, the labeled chitosan was freeze‐dried overnight, and a faint yellow powder was obtained. The same synthesis procedure as for trehalose nanoparticles was performed to obtain the FITC‐NPs.

##### Characterization of Nanoparticle and FITC‐Labeled Nanoparticles

The size and surface zeta potential of nanoparticles were assessed by Malvern Zetasizer Nano ZS90 (Worcestershire, UK). NP samples were dispersed in phosphate buffer (1 × 10^−3^ m, pH 7.4, filtered by 0.22 µm) at a concentration of around 5 mg mL^−1^ for size distribution and 1 mg mL^−1^ for surface zeta potential.

##### Transmission Electron Microscopy

The morphology of the nanoparticles was observed under a TEM. Samples were placed on Formvar 400 mesh carbon‐coated copper grid films and covered with a drop of 2% phosphotungstic acid, then imaged by TEM (FEI Technai T20, Thermo Fisher Scientific). All formulations were imaged immediately on preparation.

##### Measurement of Loading Content of Trehalose

The loading content (LC) is defined as the ratio between the weight percentage of trehalose in nanoparticles to the total weight of both trehalose and nanoparticles. It was calculated by measuring the trehalose concentration using a trehalose assay kit (Megazyme, Wicklow, Ireland) according to the manufacturer's instructions. Briefly, trehalose was phosphorylated using hexokinase and adenosine‐5′‐triphosphate (ATP) to glucose‐6‐phosphate (G6P). G6P dehydrogenase (G6P‐DH) was then used to catalyze the oxidation of G6P, in the presence of nicotinamide–adenine dinucleotide phosphate (NADP^+^), to gluconate‐6‐phosphate and a reduced form of nicotinamide–adenine dinucleotide phosphate (NADPH). The absorbance of NADPH was then measured at 340 nm using a Biotek multiple plate reader to determine the amount of trehalose in the sample.

##### Trehalose Release from Nanoparticles

Trehalose release studies from chitosan/TPP nanoparticles were performed in PBS at two pH values (5 and 7.4). About 100 mg of NPs encapsulating trehalose was suspended in 10 mL of PBS and then transferred into a dialysis bag (1000 Da MWCO). The dialysis bag was soaked in 100 mL of PBS buffer at pH 5 or 7.4 at room temperature with stirring. At various time points (1, 3, 6, 12, 24, 36, and 48 h) 1 mL of outer buffer was collected and replaced with 1 mL fresh PBS buffer. All collected samples were centrifuged and the concentration of trehalose in the supernatant was measured by the trehalose assay kit (Megazyme, Wicklow, Ireland).

##### Cellular Uptake Assay

To visualize uptake of nanoparticles by NK cells, NK‐92 cells were seeded in the wells of a 24‐well plate at the density of 5.0 × 10^4^ per well and 500 µL of nanoparticle suspension (in ddH_2_O, ≈5 mg mL^−1^) was added into the wells. Free FITC (in PBS) solution was prepared as a control. After incubation at 37 °C for 3 h, the cells were collected by centrifugation at 125 × *g* for 10 min, washed with 1× PBS three times and stained with PI (5 µg mL^−1^) for 5 min at room temperature. After washing excess stain off, the cells were transferred into a 35 mm glass‐bottom dish for imaging by Nikon A1RMP confocal microscopy.

##### Nanoparticle Toxicity to NK Cells

The effect of nanoparticles on the viability of NK cells was assessed via the CCK‐8 assay following the manufacturer's instructions. Briefly, cells were collected and seeded at a density of 2 × 10^4^ cells per well in a 96‐well plate with 100 µL medium. 10 µL of various concentrations of nanoparticles (in PBS) was added into appropriate wells to final nanoparticle concentrations ranging from 0 to 8 mg mL^−1^. The cells were incubated for 24, 48, and 72 h at 37 °C with 5% CO_2_. At predetermined exposure times, 10 µL of CCK‐8 reagent (Glpbio, Montclair, CA, USA) was added to the wells, and the cells were further incubated for 4 h at 37 °C. Absorbance (*A*) at 450 nm was measured using a microplate reader. Cell viability of each sample was calculated using the equation: Cell viability (%) = [(*A*_s_ − *A*_b_)/(*A*_c_ − *A*_b_)] × 100 (%).

##### Cryopreservation of NK Cells

To cryopreserve NK‐92 cells, the cells were seeded in wells of a 6‐well plate at a density of 1 × 10^6^ cells per well in 2 mL medium. The cells were then incubated with medium containing one of three experimental groups: free trehalose, empty chitosan/TPP nanoparticles, or nanoparticles encapsulating trehalose for 12 h. Cells incubated in medium without any additional supplement were also prepared and used as the DMSO control group. After 12 h incubation, the cells were collected and washed once with 1× PBS. Cell number and viability were measured by trypan blue staining. The collected cells were resuspended in 1 mL freezing medium; the DMSO group was resuspended in 50% FBS + 40% culture medium + 10% DMSO, while the DMSO, free trehalose, empty nanoparticles, and nTre groups were resuspended in 50% FBS + 50% culture medium containing 200 × 10^−3^ m trehalose. The cell suspensions were transferred into cryovials (Fisher Scientific, Pittsburgh, PA, USA), which were then cooled at a rate of −1 °C min^−1^ to −80 °C. The next day, the samples were transferred into liquid nitrogen. After 3 days of storage, the cryovials were removed from the liquid nitrogen tank and thawed in a 37 °C water bath. The cells were then washed with 10 mL medium for further analysis.

##### Morphological Characterization of NK Cells

To check the morphology of the recovered NK‐92 cells following cryopreservation, cells were cultured in a 6‐well plate at a density of 2 × 10^5^ cells mL^−1^. After 3 days in culture, cells were observed under an inverted microscope.

##### Viability Assay

To evaluate the viability of NK‐92 cells after cryopreservation and rewarming, the recovered cells were stained using trypan blue (Sigma–Aldrich). The live cells were counted using a hemacytometer (Thermo Fisher Scientific). The percentage of cell viability was calculated by dividing the number of live cells over the total number (live + dead) of cells.

##### NK Cell Killing Assay

To measure the killing ability of NK‐92 cells following cryopreservation, K562 target cells were used. Briefly, K562 cells were first labeled with CFSE and then seeded into a 12‐well plate with NK‐92 cells recovered, post‐thaw, from either the DMSO group or the nTre groups at E:T ratios of 1:1, 5:1, and 10:1. After incubation at 37 °C for 4 h, the cells were collected and washed twice with assay buffer, then 7‐AAD viability dye was add added to the cell suspension for 15 min at 4 °C. After staining, cells were collected and analyzed by flow cytometry on BD Fortessa Cell Analyzer (Becton Dickinson). Data were analyzed using FlowJo. A representative flow cytometry dot plot for 7‐AAD/CFSE gating is shown in Figure S5 (Supporting Information), and dot plots for killing assay controls are shown in Figure S6 (Supporting Information), while representative killing assay dot plots are shown in Figure S7 (Supporting Information).

##### IFN‐γ Release by NK Cells

CFSE‐labeled K562 and NK‐92 cells were seeded into a 12‐well plate as before. To measure the intracellular production of IFN‐γ in NK‐92 cells, Golgi‐Plug (BD Biosciences) was added to each well containing NK‐92 and K562 cells. After co‐culture for 4 h, the cells were transferred into microtubes and fixed with Cytofix/Cytoperm (BD Biosciences) for 20 min at 4 °C. Then, the cells were stained with PerCP–Cy5.5‐conjugated IFN‐γ antibody (clone B27, BD Biosciences) in Perm/Wash buffer (BD Biosciences) for 30 min at 4 °C. After washing twice with Perm/Wash buffer, the cells were collected and analyzed by flow cytometry on a BD Fortessa Cell Analyzer (Becton Dickinson). Data were analyzed using FlowJo. A representative dot plot for IFN‐γ expression is shown in Figure S8 (Supporting Information).

##### Degranulation of NK Cells

To detect the degranulation of NK cells, CD107a staining was performed. K562 cells labeled with CFSE were seeded with NK‐92 cells from the DMSO and nTre groups as before. The E:T ratio was kept at 1:1, 5:1, and 10:1. PE‐conjugated‐CD107a antibody (clone H4A3, BioLegend) was also added to each well. After 1 h, GolgiStop (BD Biosciences) was added to the cells to prevent intracellular protein transport. After an additional 3 h, the cells were collected, washed, and analyzed by flow cytometry on a BD Fortessa Cell Analyzer (Becton Dickinson). Data were analyzed using FlowJo. A representative dot plot for degranulation by CD107a is shown in Figure S9 (Supporting Information).

##### Statistical Analysis

Data were presented as mean ± standard deviation (SD). Statistical analysis was performed using Graphpad Prism 8 (the version of 8.2.1). The difference between the two groups was analyzed by a one‐way ANOVA analysis. *p* < 0.05 was considered to be statistically significant.

## Conflict of Interest

The authors declare no conflict of interest.

## Supporting information

Supporting Information
