# Supplementary material for: Nanoparticle‐Mediated Intracellular Protection of Natural Killer Cells Avoids Cryoinjury and Retains Potent Antitumor Functions
Source: Adv Sci (Weinh). 2020 Mar 13;7(9):1902938. doi: 10.1002/advs.201902938 (PMC7201255; doi:10.1002/advs.201902938)
Supplement: Supplementary file 1 — Supporting Information [file ADVS-7-1902938-s001.pdf]

Copyright WILEY-VCH Verlag GmbH & Co. KGaA, 69469 Weinheim, Germany, 2020.

ADVANCED SCIENCE
Open Access

Supporting Information

for *Adv. Sci.,* DOI: 10.1002/advs.201902938

Nanoparticle-Mediated Intracellular Protection of Natural Killer Cells Avoids Cryoinjury and Retains Potent Antitumor Functions

*Xue Yao, Joshua J. Jovevski, Michaela F. Todd, Rui Xu, Yining Li, Jiao Wang, and Sandro Matosevic**

Copyright WILEY-VCH Verlag GmbH & Co. KGaA, 69469 Weinheim, Germany, 2020.

Supporting Information

Nanoparticle-mediated intracellular protection of natural killer cells avoids cryoinjury and retains potent anti-tumor functions

Xue Yao[1], Joshua J. Jovevski[1], Michaela F. Todd[1], Rui Xu[1], YiNing Li[1], Dr. Jiao Wang[1], and Dr. Sandro Matosevic[1,2,]*

[1] Department of Industrial and Physical Pharmacy, Purdue University, West Lafayette IN, 47907 USA
[2] Center for Cancer Research, Purdue University, West Lafayette IN, 47907 USA

(A)

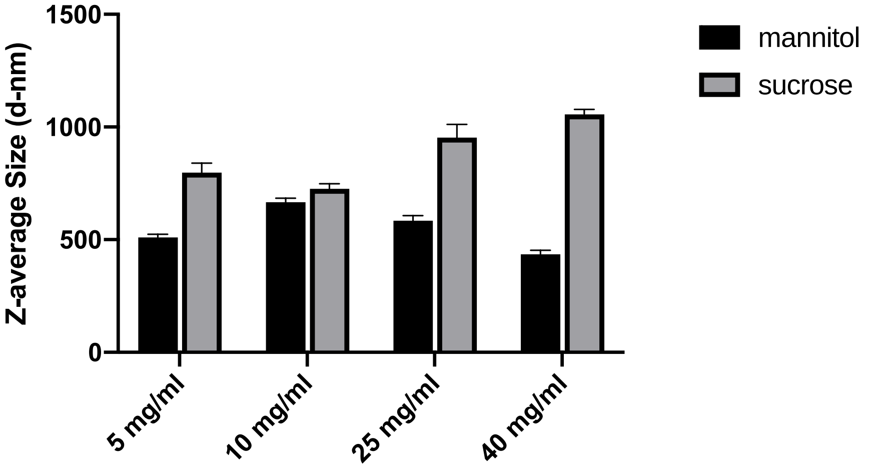

(B)

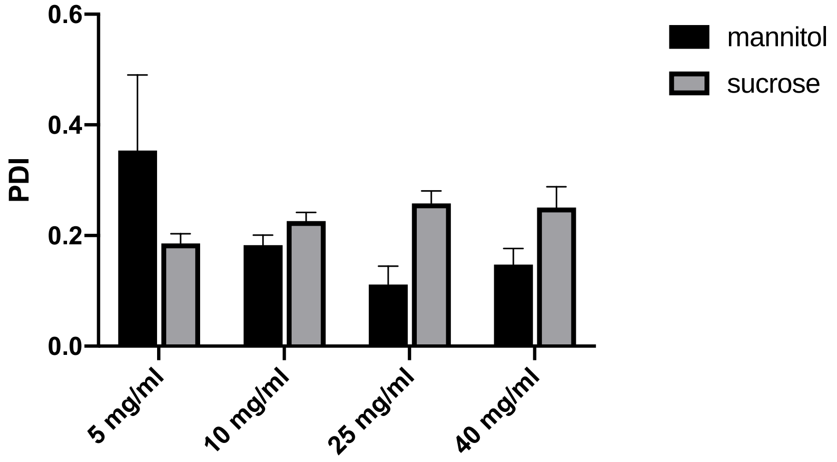

(C)

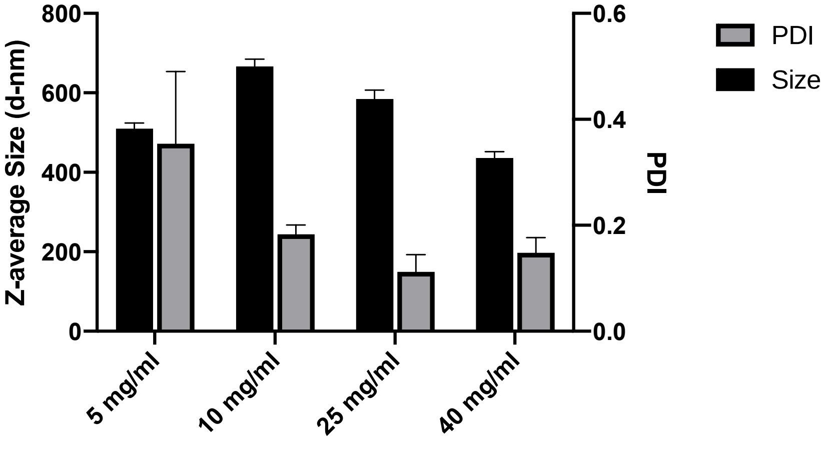

Figure S1. Effect of lyoprotectants on the size and PDI of nanoparticles. (A) Comparison of nanoparticle size after freeze-drying in the presence of either mannitol or sucrose; **(B)** Nanoparticle PDI after lyophilization with mannitol or sucrose; **(C)** Combined data for size and PDI for nanoparticles freeze-dryed in the presence of mannitol.

(A)

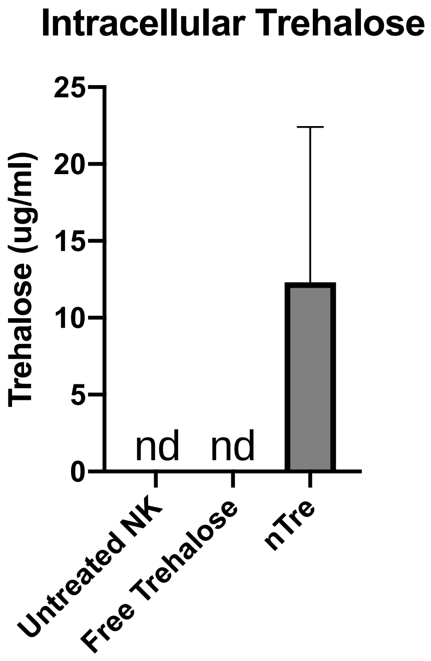

Figure S2. Release of internalized trehalose from NK cells shows successful encapsulation with no premature loss of trehalose. nTre (0.1 mM) or free trehalose were incubated with NK cells for 12 hours. Unterated NK cells were used as an additional control. After incubation, NK cells were lysed with 80% methanol solution (v/v, water, filtered through a 0.22 µm filter) at 80 °C. After air drying on a heat block, samples were re-dissolved and trehalose released from the cell was measured as before. A significant amount of trehalose released from the NK cells treated with nanoparticle and trehalose, but none was detectable (nd) for either free trehalose or untreated NK cells in any of the independent experiments. This confirms successful internalization of trehalose into NK cells. Independent experiments measured in triplicates.

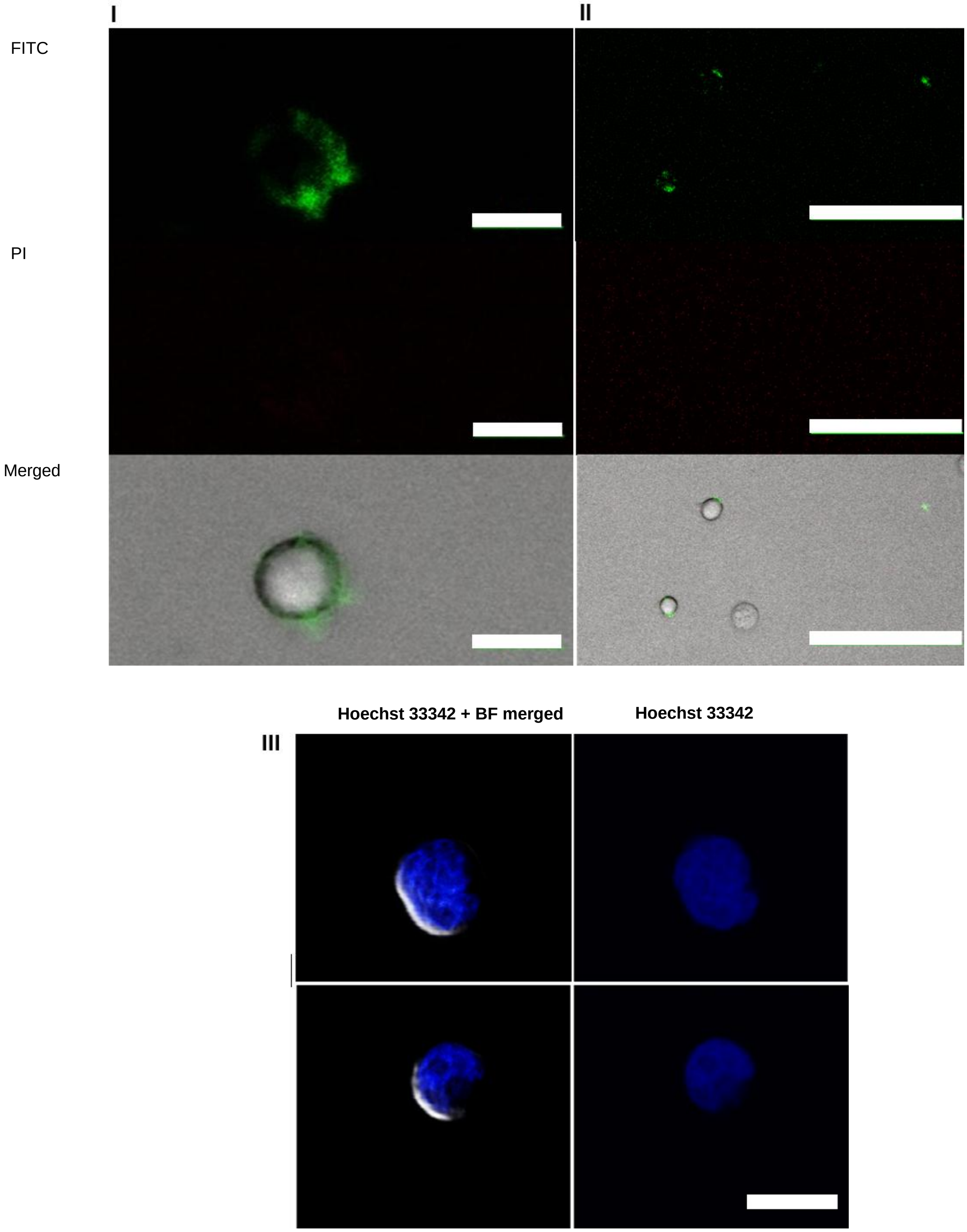

Figure S3. Intracellular NK cell and nanoparticle staining to show nanoparticle internalization. FITC labeled-nanoparticles (FITC) were incubated and internalized by NK cells. Propidium iodide (PI) was used as a stain for dead cells. NK cells were stained with Hoechst 33342 nucleic acid stain to stain the nuclei. As shown, nanoparticles localized in the narrow cytoplasmic area and not in the nucleus. The hollow center in panels corresponding to FITC-labeled nanoparticles are indicative of cytoplasmic localization. NK cells displayed a large nuclear area (*bottom row*) further confirming internalization outside of the nucleus. *BF – brightfiel; Scale bars: I and III = 10 µm; II = 100 µm*

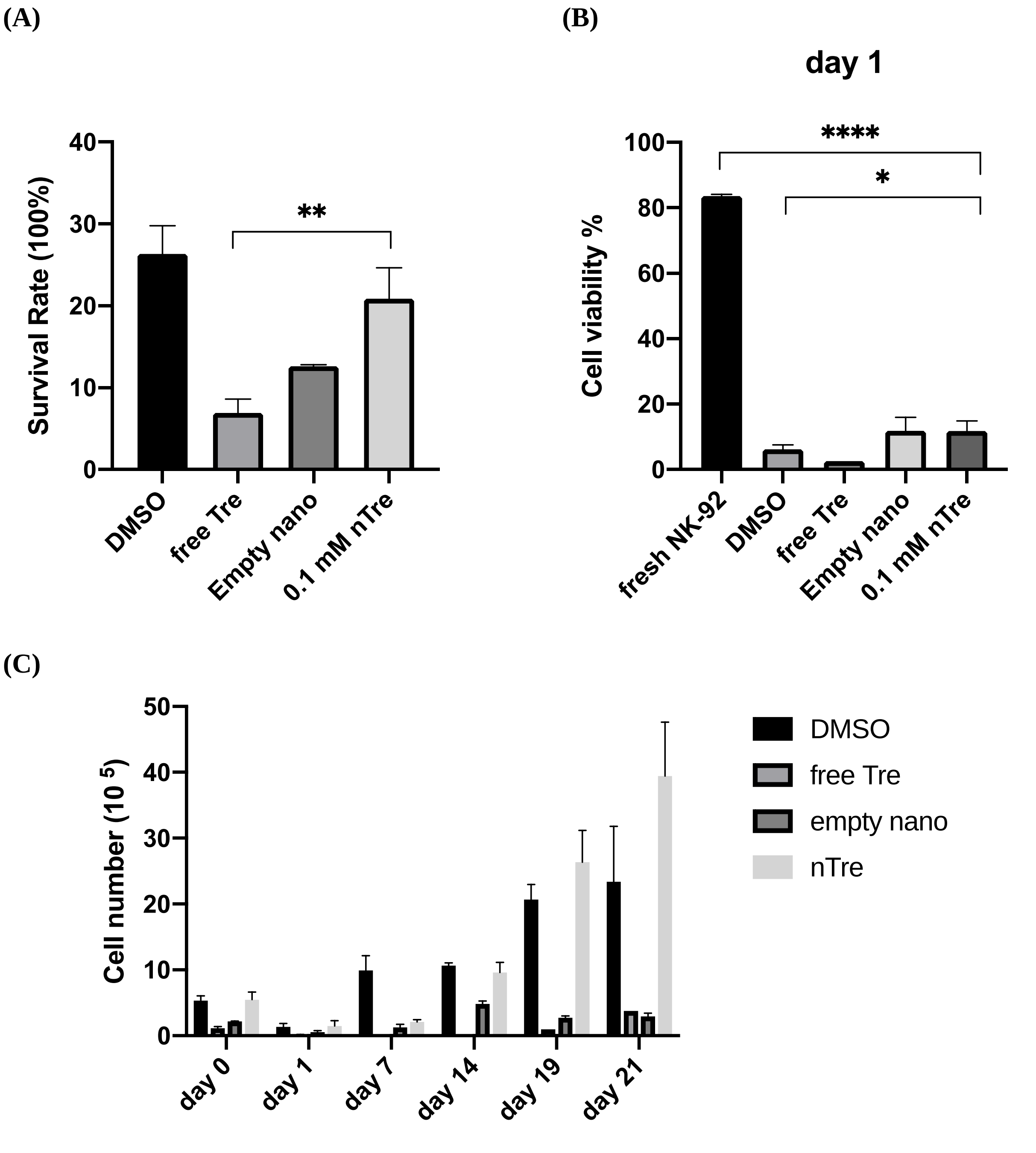

Figure S4. Characterization of cryopreserved NK cells. (A) NK cell survival rate post-thaw for cells cryopreserved with DMSO, free trehalose, empty nanoparticles and trehalose-loaded nanoparticles (nTre); **(B)** NK cell viability 24 hours after thawing; **(C)** NK cell number after thawing over 21 days in culture.

Table S1. Experimental setup for NK cell cytotoxicity assay.

	E:T 1:1	E:T 5:1	E:T 10:1	Target single	Target + CFSE	Target + 7-AAD	Target + CFSE + 7-AAD	CFSE-Target + Effector	CFSE-Target + Effector + 7-AAD
**Target cells: K562**	$4.0x10^4$/well	$4.0x10^4$/well	$4.0x10^4$/well	$4.0x10^4$/well	$4.0x10^4$/well	$4.0x10^4$/well	$4.0x10^4$/well	$4.0x10^4$/well	$4.0x10^4$/well
**Effector cells: NK92**	$4.0x10^4$/well	$2.0x10^5$/well	$4.0x10^5$/well	NA	NA	NA	NA	$4.0x10^4$/well	$4.0x10^4$/well

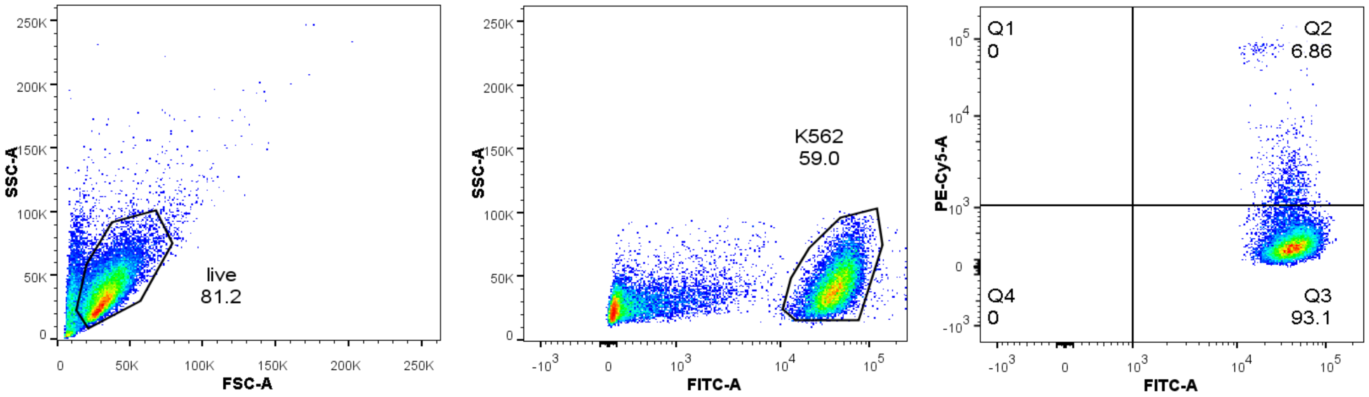

Figure S5. Gating strategy for CFSE/7-AAD killing assay.

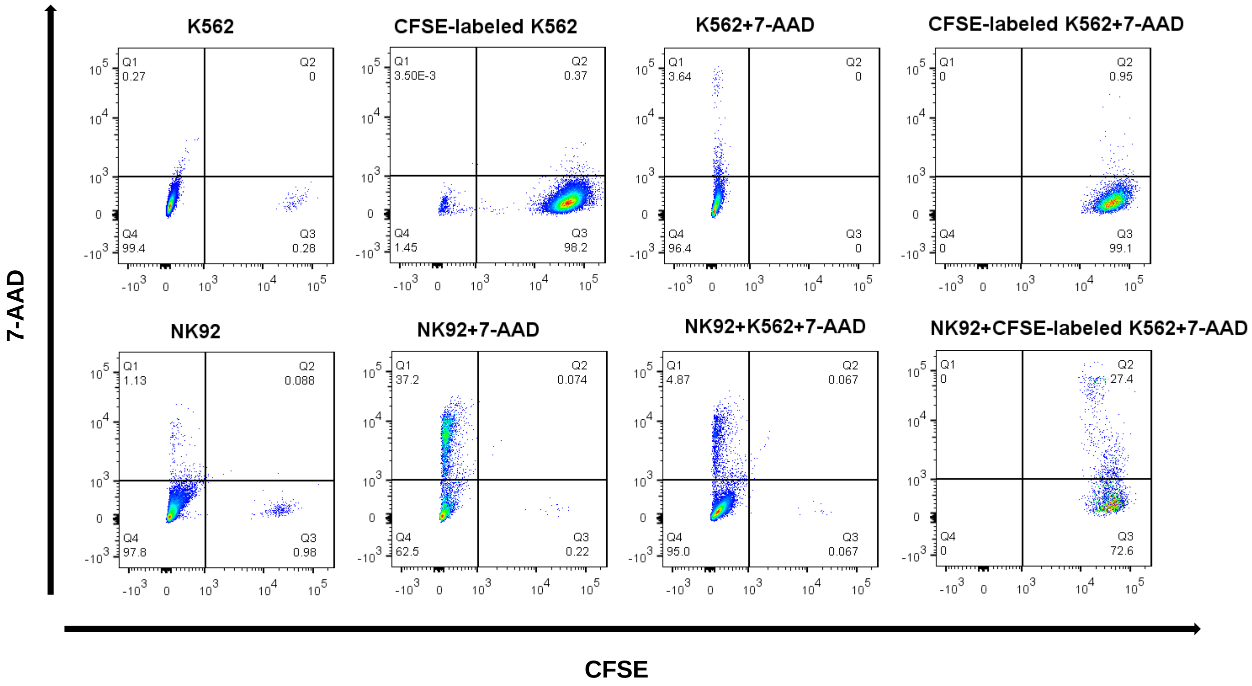

Figure S6. Flow cytometry dot-plots of killing assay controls.

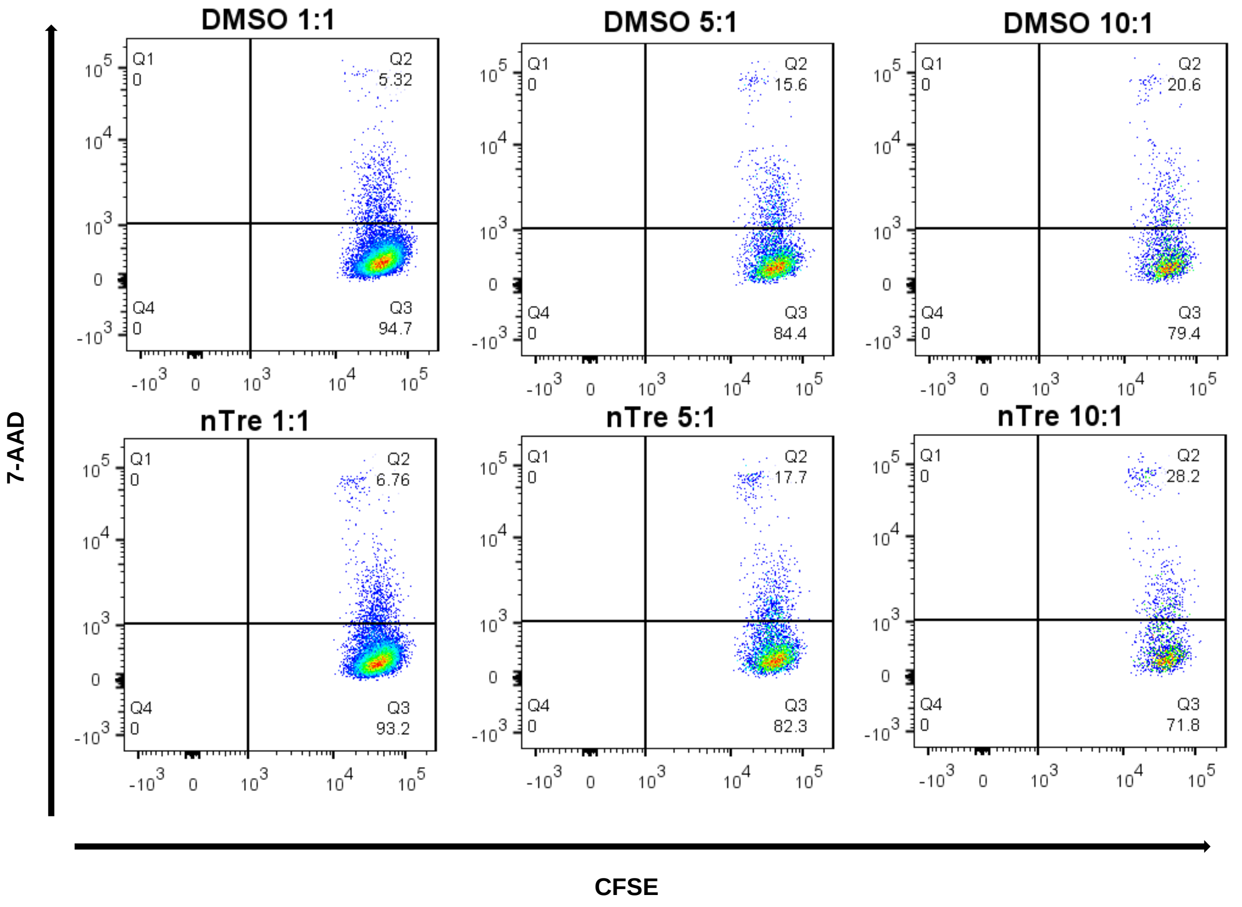

Figure S7. Representative dot-plot of NK cell killing assay.

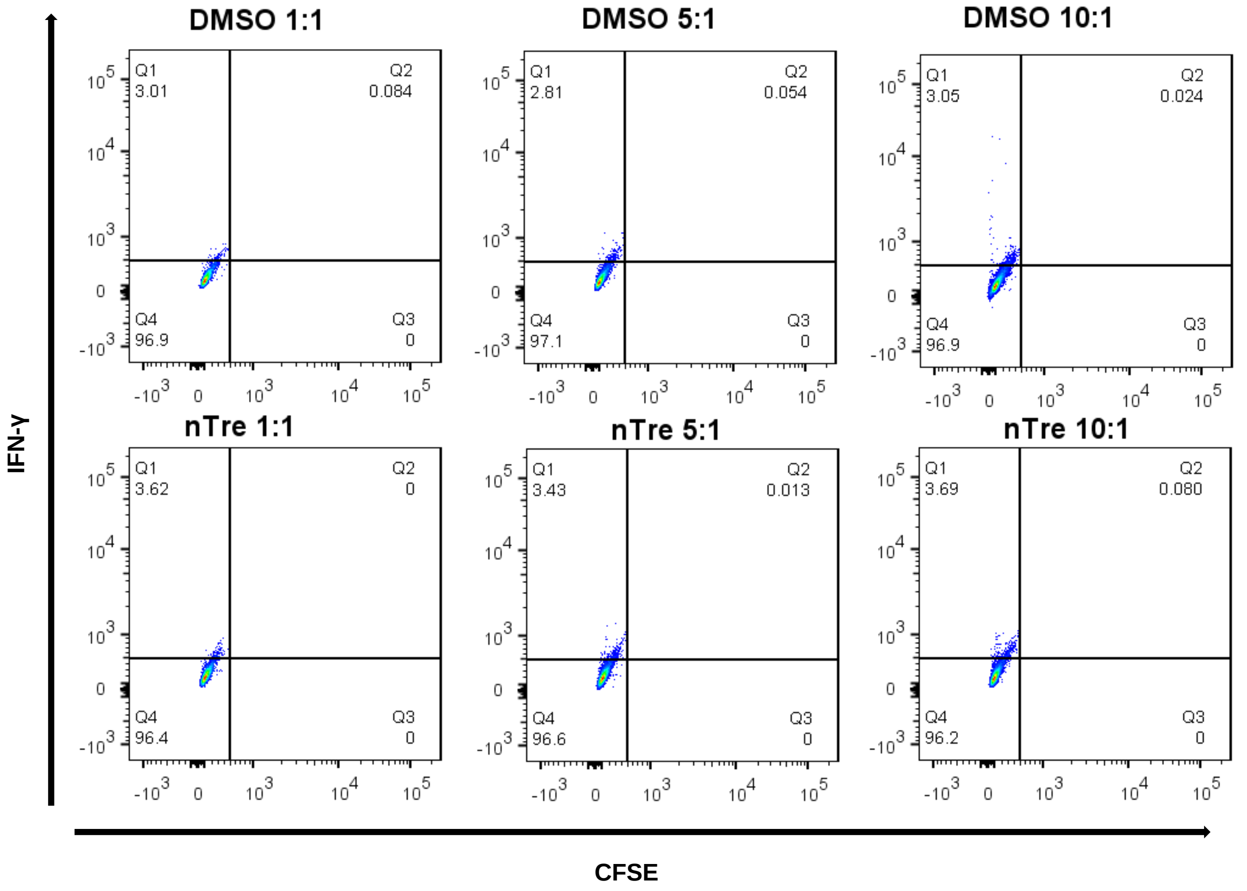

Figure S8. Representative flow cytometry dot-plots of IFN-γ expression.

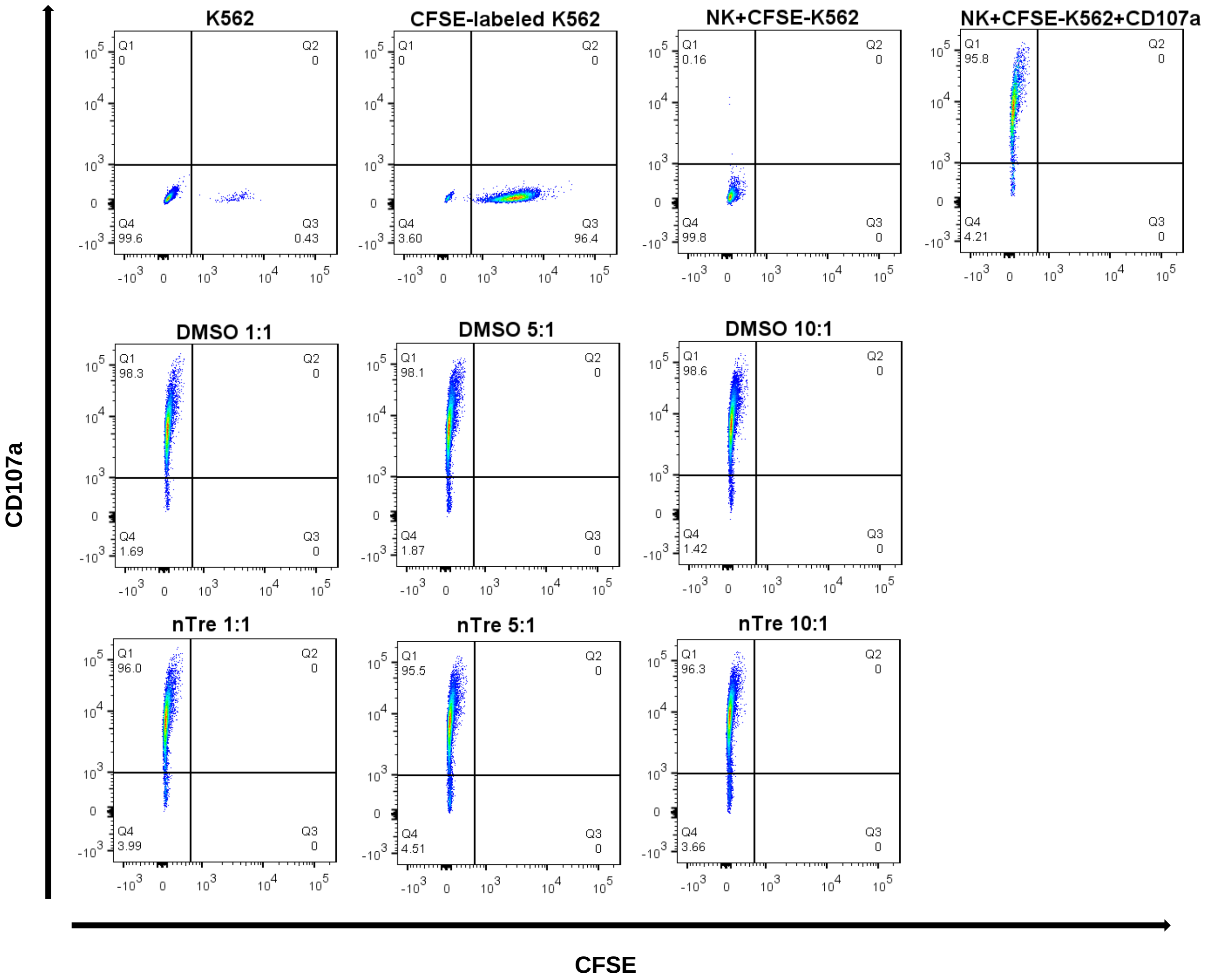

Figure S9. Representative flow cytometry dot-plots for NK cell degranulation assay.
